# Welcome to pandoraviruses at the ‘Fourth TRUC’ club

**DOI:** 10.3389/fmicb.2015.00423

**Published:** 2015-05-18

**Authors:** Vikas Sharma, Philippe Colson, Olivier Chabrol, Patrick Scheid, Pierre Pontarotti, Didier Raoult

**Affiliations:** ^1^Unité de Recherche sur les Maladies Infectieuses et Tropicales Emergentes UM63 CNRS 7278, IRD 198, INSERM U1095, Faculté de Médecine, Aix-Marseille UniversityMarseille, France; ^2^I2M UMR 7373, CNRS équipe Evolution Biologique et Modélisation, Aix-Marseille UniversityMarseille, France; ^3^Institut Hospitalo-Universitaire Méditerranée Infection, Pôle des Maladies Infectieuses et Tropicales Clinique et Biologique, Fédération de Bactériologie-Hygiène-Virologie, Centre Hospitalo-Universitaire Timone, Assistance Publique-Hôpitaux de MarseilleMarseille, France; ^4^Medical Parasitology Laboratory, Laboratory Department I (Medicine), Diagnostics, Central Institute of the Bundeswehr Medical ServiceKoblenz, Germany

**Keywords:** giant virus, *Pandoravirus*, nucleocytoplasmic large DNA viruses, *Megavirales*, informational genes, domains of life, TRUC, phylogeny

## Abstract

Nucleocytoplasmic large DNA viruses, or representatives of the proposed order *Megavirales*, belong to families of giant viruses that infect a broad range of eukaryotic hosts. Megaviruses have been previously described to comprise a fourth monophylogenetic TRUC (things resisting uncompleted classification) together with cellular domains in the universal tree of life. Recently described pandoraviruses have large (1.9–2.5 MB) and highly divergent genomes. In the present study, we updated the classification of pandoraviruses and other reported giant viruses. Phylogenetic trees were constructed based on six informational genes. Hierarchical clustering was performed based on a set of informational genes from *Megavirales* members and cellular organisms. Homologous sequences were selected from cellular organisms using TimeTree software, comprising comprehensive, and representative sets of members from *Bacteria, Archaea,* and *Eukarya*. Phylogenetic analyses based on three conserved core genes clustered pandoraviruses with phycodnaviruses, exhibiting their close relatedness. Additionally, hierarchical clustering analyses based on informational genes grouped pandoraviruses with *Megavirales* members as a super group distinct from cellular organisms. Thus, the analyses based on core conserved genes revealed that pandoraviruses are new genuine members of the ‘Fourth TRUC’ club, encompassing distinct life forms compared with cellular organisms.

## Introduction

Defining microbes has been a long journey. During the 18th century, Pasteur first described these organisms as microscopic organisms invisible to the naked eye, but visible under the microscope (Raoult, 2013). At the beginning of the 20th century, Chatton classified these microbes as eukaryotes and prokaryotes based on the presence or absence of a nucleus, and C. Woese subsequently classified these organisms into three domains of life, *Archaea*, *Bacteria,* and *Eukarya*, based on ribosomal DNA (Woese et al., 1990; Raoult, 2013). Although recently revealed as the most abundant biological entities in the biosphere, infecting cellular organisms from these three domains, viruses have been excluded from this classification of the living world because these organisms lack ribosomal DNA (Suttle, 2005; Raoult and Forterre, 2008; Raoult, 2013, 2015). Indeed, viruses were defined as invisible under a light microscope, and these organisms were initially referred to as inframicrobes. However, giant viruses of amoeba, first described in 2003 with the discovery of Mimivirus as a *bona fide* microbe, have suggested that this paradigm should be revisited (Raoult et al., 2004). Mimivirus, which was isolated through co-culturing with *Acanthamoeba polyphaga*, was initially considered to be a small bacterium due to its large size (750 nm in diameter) and Gram-positive staining (Raoult et al., 2007). Mimivirus has a large genome comprising 1.2 megabase pairs (Mbp) that was until recently the largest amongst viruses and even larger than those of small bacteria, such as *Mycoplasma genitalium* (Raoult et al., 2004). Moreover, this microbe has a repertoire of almost 1,000 genes encoding unexpected proteins, previously considered to be a trademark of cellular organisms, including components of the translation apparatus. Subsequently, several dozens of giant viruses of amoeba have been identified and classified in the family *Mimiviridae*, founded by Mimivirus (La Scola et al., 2008; Fischer et al., 2010; Arslan et al., 2011; Yoosuf et al., 2012; Santini et al., 2013; Yutin et al., 2013), or family *Marseilleviridae*, founded by Marseillevirus (Boyer et al., 2009; Thomas et al., 2011; Pagnier et al., 2013; Aherfi et al., 2014). These viruses are commonly observed in the biosphere, consistent with the frequent isolation of these organisms from environmental water, and sediment/soil samples in several countries and the concurrent detection of sequences related to the genomes of these microbes in various environmental metagenomes (Ghedin and Claverie, 2005; Monier et al., 2008; Kristensen et al., 2010; Williamson et al., 2012). Moreover, mimiviruses and marseilleviruses have been recently identified in human samples, associated with pneumonia and adenitis, respectively, (Popgeorgiev et al., 2013; Saadi et al., 2013). Giant viruses of amoeba have been associated with nucleocytoplasmic large DNA viruses (NCLDVs), a monophyletic group of viruses first identified in 2001, encompassing asfarviruses, poxviruses, phycodnaviruses, ascoviruses, and iridoviruses (Iyer et al., 2001). The reclassification of all NCLDV families in a new viral order, called the *Megavirales*, has recently been proposed (Colson et al., 2012, 2013). Phylogenetic and phyletic analyses have revealed a common origin for these viruses dating back to the early stage of eukaryotic evolution (Yutin et al., 2009; Boyer et al., 2010; Koonin and Yutin, 2010; Yutin and Koonin, 2012).

Subsequent to the identification of Mimivirus, the idea of a fourth domain of life emerged based on the phylogeny of the conserved genes shared by this virus and members from *Eukarya*, *Archaea,* and *Bacteria* (Raoult et al., 2004). Subsequently, in 2010, phylogenetic and phyletic analyses of informational genes involved in nucleotide metabolism and DNA processing and shared by cellular organisms and giant viruses suggested that the *Megavirales* members comprise a fourth branch of life with an origin as ancient as that of the three cellular branches (Boyer et al., 2010). This four branch topology was criticized based on the argument that the single-matrix evolutionary models used by Boyer et al. (2010) for their phylogeny reconstructions were not adequate with respect to the substantial compositional heterogeneity bias and homoplasy detected in the informational genes used (Williams et al., 2011). Accordingly, alternative trees were proposed by Williams et al. (2011) using models presented as more appropriate, but these phylogeny reconstructions did not show a monophyly for *Eukarya*. Besides, it was proposed that the informational genes present in *Megavirales* members might have been acquired from eukaryotic hosts through horizontal gene transfer (Moreira et al., 2005; Moreira and Brochier-Armanet, 2008; Yutin et al., 2014). In contrast, other findings have strengthened the fourth branch of life assumption. Thus, phylogeny reconstructions describing the evolution of proteomes and conserved protein domain structures of cellular organisms and giant viruses suggested that megaviruses comprise a fourth super group that is distinct from the *eukaryotic, archaeal, and bacterial* groups in the universal tree of life (Nasir et al., 2012). In addition, RNA polymerase (RNAP) beta subunit homologs were detected in the Global Ocean Sampling (GOS) expedition metagenomic database, representing novel branches, apart from those encompassing *Bacteria*, *Archaea,* and *Eukarya* and large viruses (only poxviruses being analyzed) and were considered to be possibly derived from unknown viruses (Wu et al., 2011). Moreover, we recently extended our 2010 study using DNA-dependent RNA polymerase subunit 2 (RNAP2) from a comprehensive, representative, and unbiased set of species from cellular organisms selected by using TimeTree, and from the *Megavirales* representatives, and the results strongly supported a four branch topology (Sharma et al., 2014). The name ‘TRUC’ (an acronym for “Things Resisting Uncompleted Classification”) corresponds to a new classification that has been recently created to accommodate the division of currently known microbes in four branches, i.e., bacteria, archaea, eukaryotes, and giant viruses (Raoult, 2013). The redefinition of microbes as TRUCs emphasizes that the three domains of life topology is based on ribosomal DNA and is thus associated with cellular organisms, while neglecting giant viruses.

In 2013, two new giant viruses, *Pandoravirus dulcis, and P. salinus*, were isolated from *Acanthamoeba* present in the mud of a pond in Australia and a marine sediment layer in Chile, respectively (Philippe et al., 2013). These viruses became the record holders of size of virions (ovoids in shape and ≈1 μm long) and contained the largest genome sizes (1.9 and 2.5 Mbp, respectively) identified to date. The genome from another *Pandoravirus* strain named *Pandoravirus inopinatum* has been recently sequenced. It is 2.2 Mbp large, being the second largest viral genome known to date, and is predicted to encode 1,902 proteins (Antwerpen et al., 2015). *P. inopinatum* was first found as an endocytobiont of *Acanthamoeba* strains isolated from the contact lens storage cases of a patient presenting keratitis, demonstrating that humans are exposed to these giant viruses (Scheid et al., 2014; Antwerpen et al., 2015). Notably, similar to Mimivirus, which has long been considered a bacterium instead of a virus (Raoult et al., 2007), *Pandoravirus*-like particles have been initially classified in 2008 as “extraordinary” endocytobionts of *Acanthamoeba* sp. and were described subsequently in greater details (Scheid et al., 2008, 2010, 2014; Scheid, 2014; Antwerpen et al., 2015). Noteworthy, *Pandoravirus*-related sequences have been recently detected in metagenomes generated from various soil samples worldwide (Kerepesi and Grolmusz, 2015).

Pandoraviruses have been identified as highly divergent viral entities. Indeed, only 16% of the predicted genes from *P. salinus* have significant matches in the NCBI GenBank sequence database, and more than half of these genes belong to families of paralogs (Philippe et al., 2013). In addition, only 14 of the 31 class I-III core genes of *Megavirales* members and 17 of the 49 inferred *Megavirales* ancestral genes have been identified in pandoraviral genomes (Philippe et al., 2013; Yutin and Koonin, 2013). Moreover, the size and shape of pandoraviruses are unique among viruses, and no capsid protein-encoding gene has been identified (Philippe et al., 2013). In the present study, we aimed to demonstrate that the giant pandoraviruses are members of the fourth TRUC.

## Materials and methods

### Viral Genes Used for the Analyses

In the present study, we used the approach previously described by Boyer et al. (2010). The genes considered herein were identified from clusters of orthologous groups of proteins (COGs) function categories associated with nucleotide transport and metabolism and information storage and processing (F, J, A, K, L, and B). Notably, these clusters included the genes encoding ribonucleotide reductase (RNR) and thymidylate synthase (TS), which are both key enzymes involved in the RNA–DNA shift; DNA polymerase family B (DNAPol); topoisomerase II A (TopoIIA); the Flap endonuclease (FEN); the processing factor Proliferating Cell Nuclear Antigen (PCNA); the RNAP2; the transcription factor II B (TFIIB); four amino-acyl tRNA synthetases; and the putative elongation factor EF-1. The presence of these genes was assessed in the genomes of pandoraviruses. Despite having tremendous gene contents, pandoraviruses lack many ancestral genes and have been described to share only 17 of the 49 conserved genes assigned to the putative common ancestor of the *Megavirales* members using maximum likelihood (ML) evolutionary reconstruction (Yutin et al., 2009) and 5 of the 12 genes involved in DNA processing. Therefore, the gene markers used in the present study included RNAP1, RNAP2, DNApol, RNR, tyrosyl-tRNA synthetase, and TFIIB. The viral orthologous sequences were collected using the OrthoMCL procedure (Li et al., 2003) with gene repertoires from 317 DNA viral genomes encoding proteomes comprising more than 100 protein sequences directly downloaded from the NCBI GenBank sequence database^1^. Recently available sequences from *P. inopinatum* were collected from the NCBI GenBank protein sequence database^2^. Comparative analysis of the gene repertoires from the three *Pandoravirus* isolates was performed using OrthoMCL and through the strategy of reciprocal best BLASTp hits (using as thresholds an *e*-value <1e-3, an amino acid identity >30% and a sequence coverage >70%) to identify the sets of *bona fide* orthologous genes (Jordan et al., 2002; Li et al., 2003).

### Search for Homologous Sequences in Cellular Life Forms

Protein BLAST searches were performed using Standalone Blast 2.2.27 (Altschul et al., 1990) and giant viral protein sequences as queries against the NCBI GenBank non-redundant protein sequence database (nr) to collect homologous sequences from the members of the three cellular domains of life (*Bacteria*, *Archaea,* and *Eukarya*). The number of target sequences was limited to 20,000 per query.

### Sequence Selection Criteria for Cellular Domains

Conserved genes might possess multiple homologs in sequence databases, and random selection among these genes for phylogeny reconstruction might result in the selection of strongly biased non-representative sequence sets. Hence, we selected homologous sequences from various species corresponding to representatives from different phyla of *Bacteria*, *Archaea,* and *Eukarya* using TimeTree, as previously described (Sharma et al., 2014). TimeTree is a public knowledge-base of divergence times among organisms estimated from molecular data in published studies (Hedges et al., 2006). This resource facilitated here the selection of sequences from species that diverged approximately 500 million years ago, which allowed obtaining an appropriate set of members from the three cellular domains of life through a good equilibrium between comprehensiveness and representativeness. In addition, the genomes of most of these cellular organisms are available and have been annotated. The Taxon filter program was subsequently used to filter out taxon and gi identification numbers from the results of the BLAST analysis, which facilitated the downloading of selected protein sequences directly from the NCBI GenBank non-redundant database. Partial or identical sequences were removed through clustering using the CD-HIT suite program (Huang et al., 2010).

### Multiple Sequence Alignments and Phylogeny Reconstructions

The obtained sequences were aligned using the Muscle program (Edgar, 2004). Alignment quality was manually analyzed, and phylogenetic reconstructions were performed using ML inference, including the WAG model, and confidence values were calculated using the Shimodaira-Hasegawa (SH) test through the FastTree program (Price et al., 2010). Phylogenetic trees were constructed using the FigTree software program^3^.

### Phyletic Pattern Analysis with Clusters of Orthologous Groups of Protein

Clusters of orthologous groups of proteins corresponding only to the selected functional COG categories [J, A, K, L, B, and F], encoding proteins involved in information storage and processing and nucleotide transport and metabolism, were used for the analysis. BLASTp searches for the selected COG categories, with *e*-values <1e-3, were performed against the members from *Bacteria*, *Archaea*, *Eukarya,* and *Megavirales*. Using the BLASTp output results, presence (1) and absence (0) matrices were constructed. A hierarchical clustering dendrogram was constructed using the Pearson distance method with R package ‘amap’^4^.

## Results

A comprehensive search for the 12 informational genes used in a previous study to delineate a fourth TRUC showed that only four genes were conserved in pandoraviruses (Boyer et al., 2010). These genes encode RNAP1 and RNAP2, a DNApol, a RNR, and a tyrosyl-tRNA synthetase.

### Phylogenetic Analyses

#### Informational Genes Showing Vertical Evolution

**FIGURE 1:**
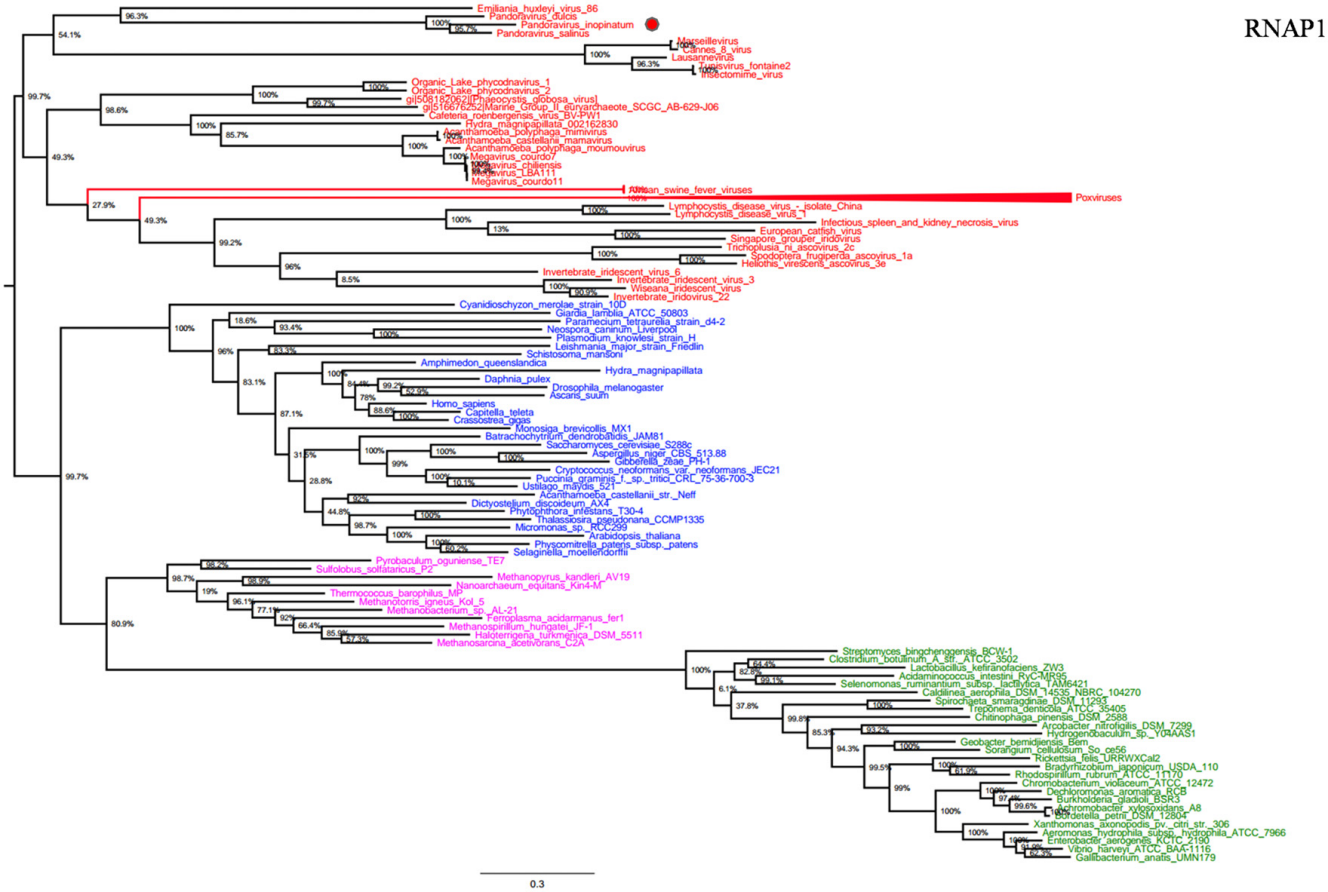
**RNA polymerase subunit 1 (RNAP1) phylogenetic tree.** RNAP1 Maximum-Likelihood (ML) tree was constructed using aligned sequences from *Megavirales* (red), *Bacteria* (green), *Archaea* (pink), *and Eukarya* (blue). The confidence values were computed through SH-like support using the FastTree program. The scale bar represents the number of estimated changes per position.

**FIGURE 2:**
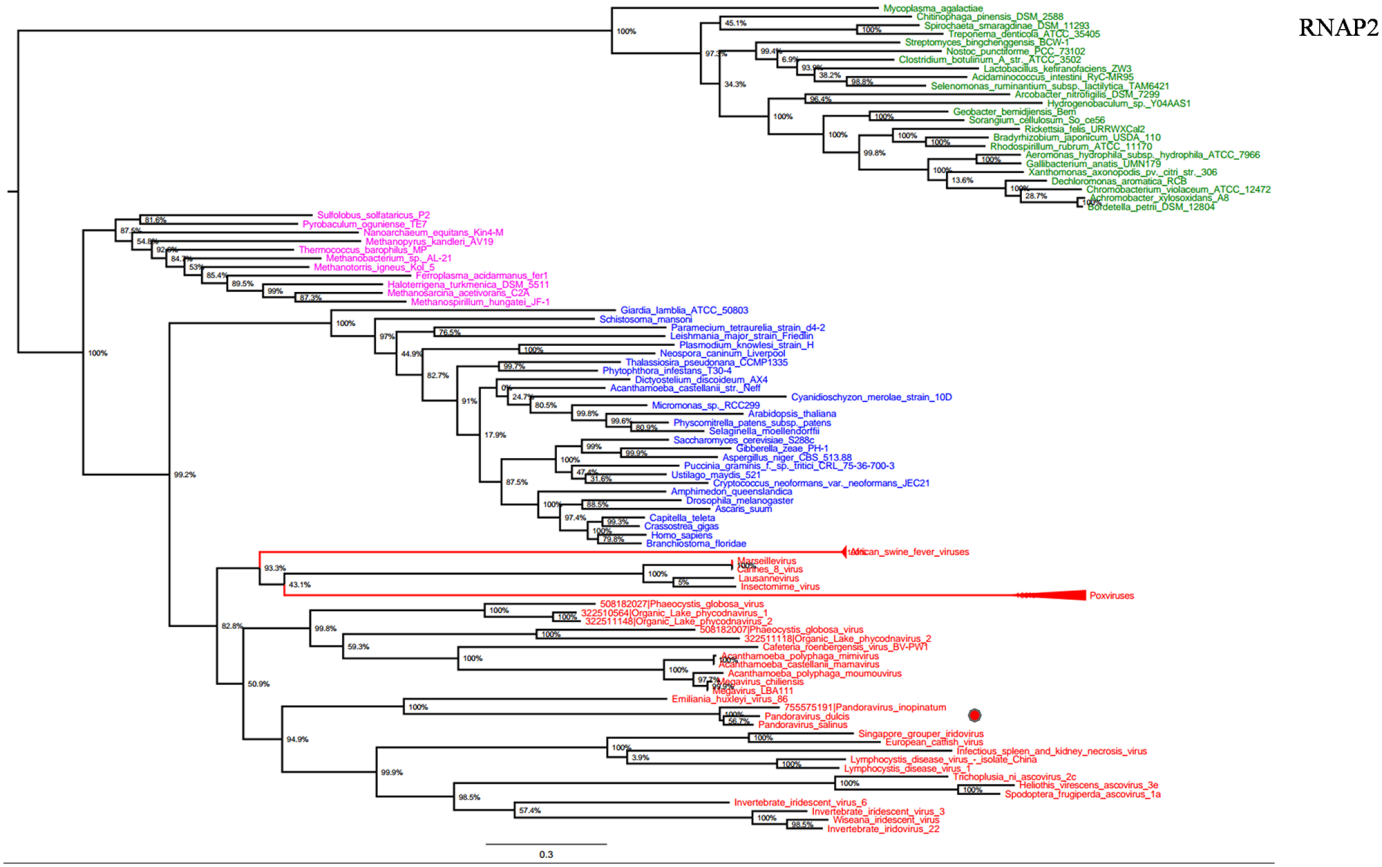
**RNAP2 phylogenetic tree.** RNAP1 ML tree was constructed using aligned sequences from *Megavirales* (red), *Bacteria* (green), *Archaea* (pink), *and Eukarya* (blue). The confidence values were computed through SH-like support using the FastTree program. The scale bar represents the number of estimated changes per position.

RNA polymerase is a multi-subunit enzyme that uses a DNA template for the synthesis of RNA to complete transcription (Werner and Grohmann, 2011). Both RNAP 1 and 2 subunits are universal informational genes conserved in all cellular life forms and *Megavirales* members (Sharma et al., 2014). Phylogenetic analyses based on RNAP1/2 showed that *P. salinus*, *P. dulcis,* and *P. inopinatum* were clustered with *Emiliana huxlei* virus, a coccolithovirus from the family *Phycodnaviridae* (**Figures 1** and **2**). Notably, newly sequenced mimiviruses or marseilleviruses, including LBA111 virus (Saadi et al., 2013), Insectomime virus (Boughalmi et al., 2013), and Tunisvirus (Aherfi et al., 2014), were identified as new *bona fide* members of the ‘Fourth TRUC’ club. Similarly, two hidden *Megavirales* members, recently misclassified as *Hydra magnipapillata*, which is a freshwater cnidarian, and a Marine Group II euryarchaeota, which is a marine archaea, were also identified as members of the ‘Fourth TRUC’ club (Sharma et al., 2014). Furthermore, RNAP1/2-based phylogeny reconstructions showed *Megavirales* and the three other cellular TRUCs as clearly distinct monophylogenetic groups.

**FIGURE 3:**
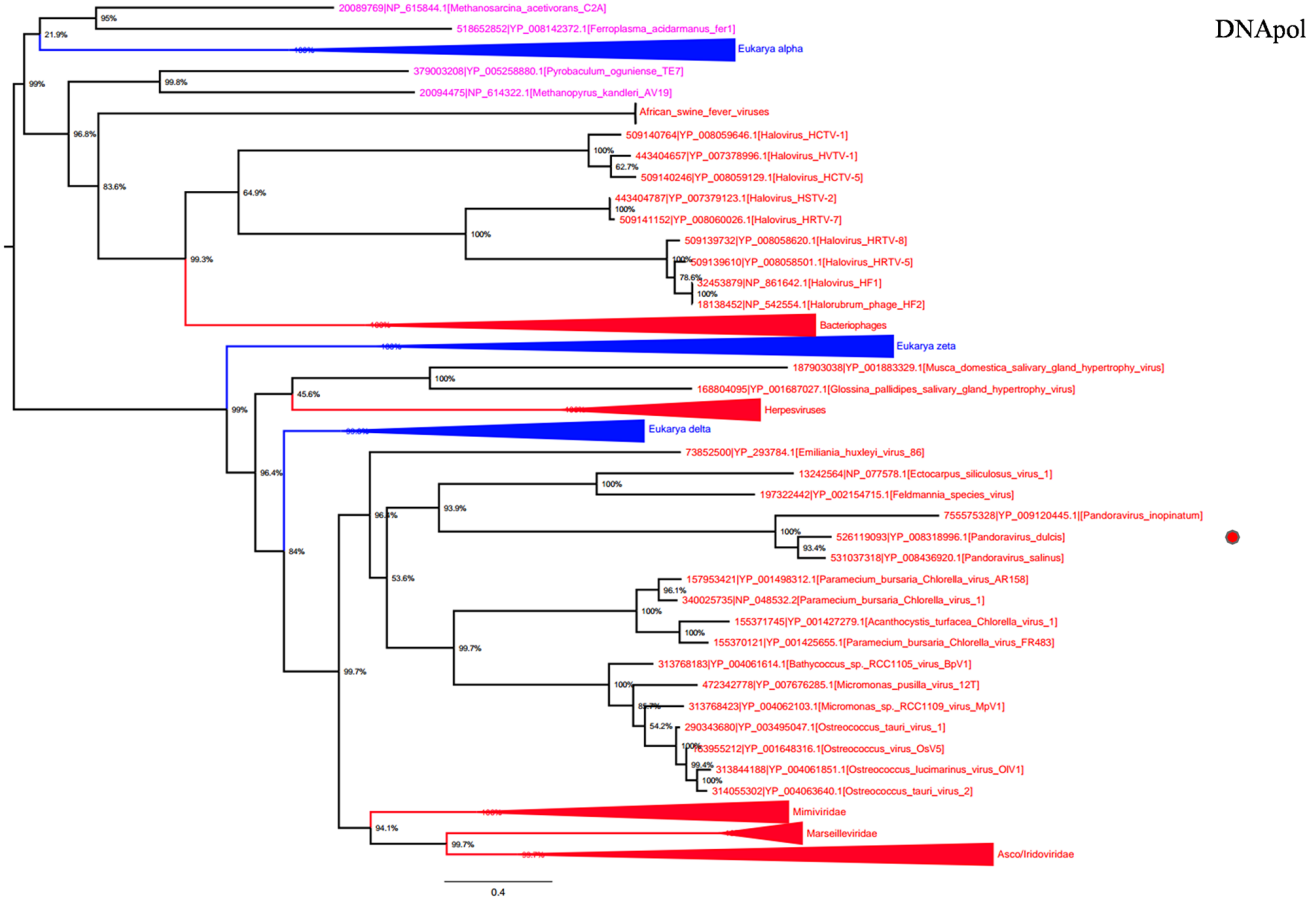
**DNA polymerase family B phylogenetic tree.** DNA polymerase family B ML tree was constructed using aligned sequences from *Megavirales* (red), *Bacteria* (green), *Archaea* (pink), *and Eukarya* (blue). The confidence values were computed through SH-like support using the FastTree program. The scale bar represents the number of estimated changes per position.

DNA polymerase family B is another highly conserved informational gene involved in DNA replication. Phylogenetic analyses based on DNApol showed that *Megavirales* members form a monophylogenetic group, with the exception of African swine fever viruses, the sole members of the family *Asfarviridae* (Dixon et al., 2000; **Figure 3**). Sequences from the three *Pandoravirus* genomes are located in the same position that in the RNAP trees, being clustered with *Emiliana huxlei* virus, a phycodnavirus. This result is congruent with those from previously described phylogeny reconstructions (Philippe et al., 2013; Yutin and Koonin, 2013) and suggests that pandoraviruses might be derived from phycodnaviruses, as previously pointed out based on phylogenomic analyses (Yutin and Koonin, 2013).

Although absent from bacteria, transcription factor TFIIB, one of the transcription factors comprising the RNA polymerase II preinitiation complex, is present in eukaryotes, archaea, and some *Megavirales* members but absent from *Pandoravirus* strains. Phylogenetic analysis based on this gene showed that the other megaviruses form a monophyletic group distinct from eukaryotic and archaeal lineages (Supplementary Figure S1).

#### Informational Genes Showing Horizontal Evolution

**FIGURE 4:**
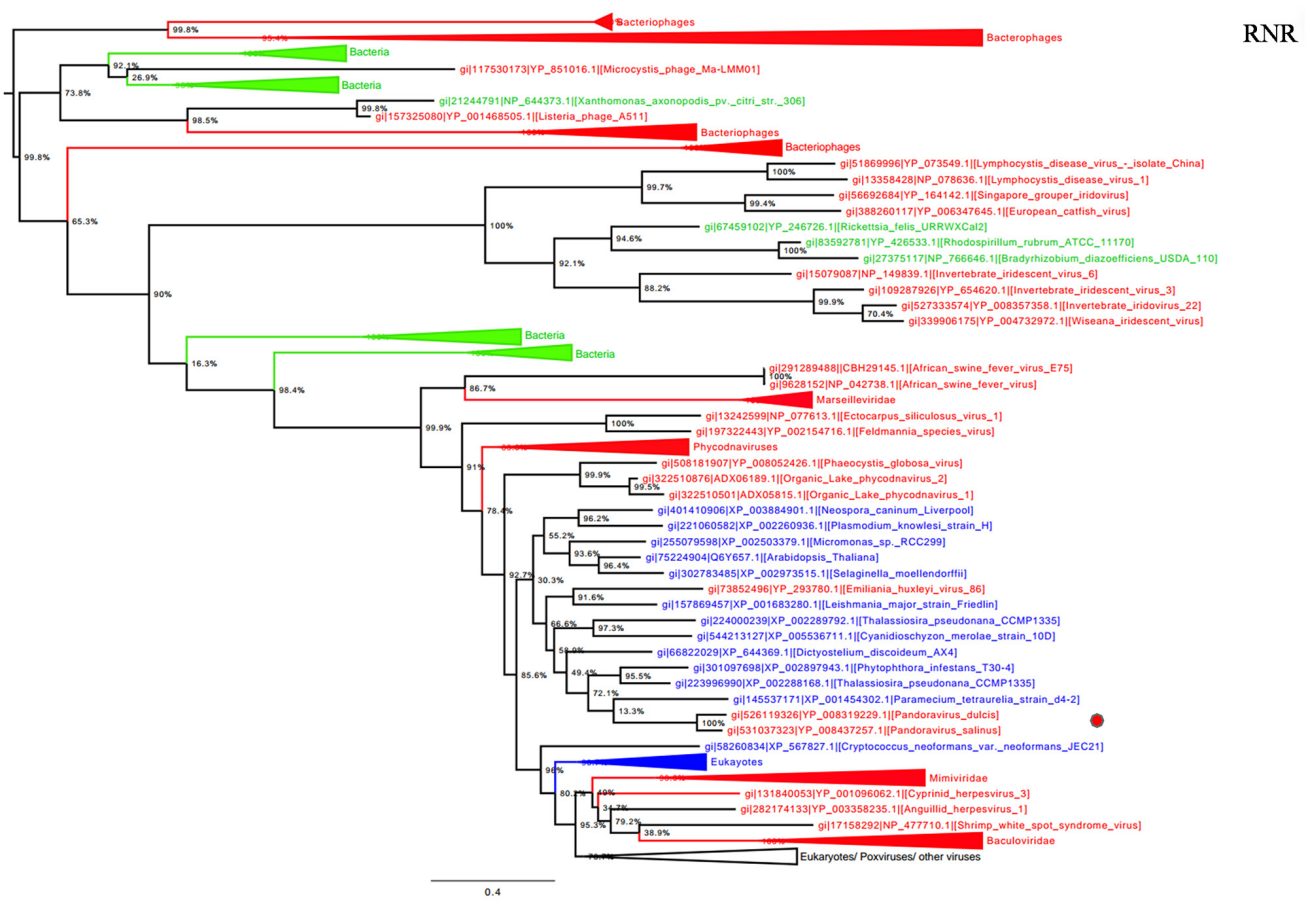
**Ribonucleotide reductase (RNR) small subunit phylogenetic tree.** RNR ML tree was constructed using aligned sequences from *Megavirales* (red), *Bacteria* (green), *Archaea* (pink), and *Eukarya* (blue). The confidence values were computed through SH-like support using the FastTree program. The scale bar represents the number of estimated changes per position.

Ribonucleotide reductase is central to the biosynthesis of DNA precursors and catalyzes the reduction of RNA precursors into dNTP (Crona et al., 2013). This gene is present in all cellular organisms and *Megavirales* members. Among pandoraviruses, only *P. inopinatum* was found to lack this gene. Phylogenetic analysis showed that *Pandoravirus* RNRs were clustered with those of eukaryotes including the soil-living amoeba *Dictyostelium discoideum* AX4, and deeply branched inside the eukaryotic clade (**Figure 4**). Interestingly, this amoeba was previously identified, unlike most eukaryotes, as encoding both a class I and II RNRs (Crona et al., 2013). These results suggest that pandoraviruses might have acquired this gene through an ancient transfer from an eukaryotic host. In addition, giant viral RNR sequences were scattered in the tree, which may suggest several independent horizontal gene transfer events involving members of the fourth TRUC. Beside this most parsimonious explanation, other evolutionary scenarios including homoplasy, and divergent evolution could not be ruled out.

**FIGURE 5:**
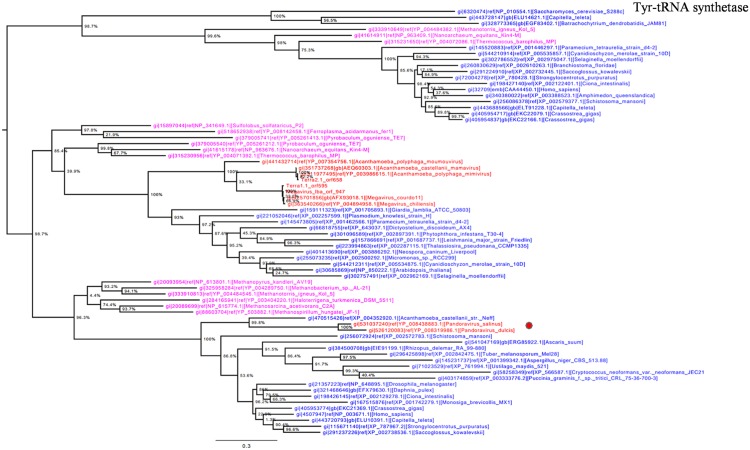
**Tyrosyl-tRNA synthetase phylogenetic tree.** Tyrosyl t-RNA ML tree was constructed using aligned sequences from *Megavirales* (red), *Bacteria* (green), *Archaea* (pink), and *Eukarya* (blue). The confidence values were computed through SH-like support using the FastTree program. The scale bar represents the number of estimated changes per position.

Otherwise, *Pandoravirus* genomes harbor several genes involved in translation, a feature shared with other giant viruses. Notably, amino-acyl tRNA synthetases are conserved in some *Megavirales* members including pandoraviruses. Tyrosyl-tRNA synthetase is present in *P. salinus* and *P. dulcis* but absent in *P. inopinatum.* Phylogeny reconstruction based on this protein showed that pandoraviruses were clustered with *Acanthamoeba castellanii* str. Neff, as observed previously (Philippe et al., 2013), suggesting that these viruses have acquired this gene from their hosts, whereas family *Mimiviridae* forms a separate monophylogenetic cluster (**Figure 5**). We did not find a clustering between an *A. castellanii* gene and a megaviral gene in phylogenies built here apart from tyrosyl-tRNA from this amoeba and from pandoraviruses. Moreover, phylogeny based on tyrosyl-tRNA synthetase provided a complex evolutionary scenario, as *Eukarya* and *Archaea* members were scattered in the tree, suggesting that horizontal gene transfers occurred several times within different phyla.

### Phyletic Pattern Analyses

**FIGURE 6:**
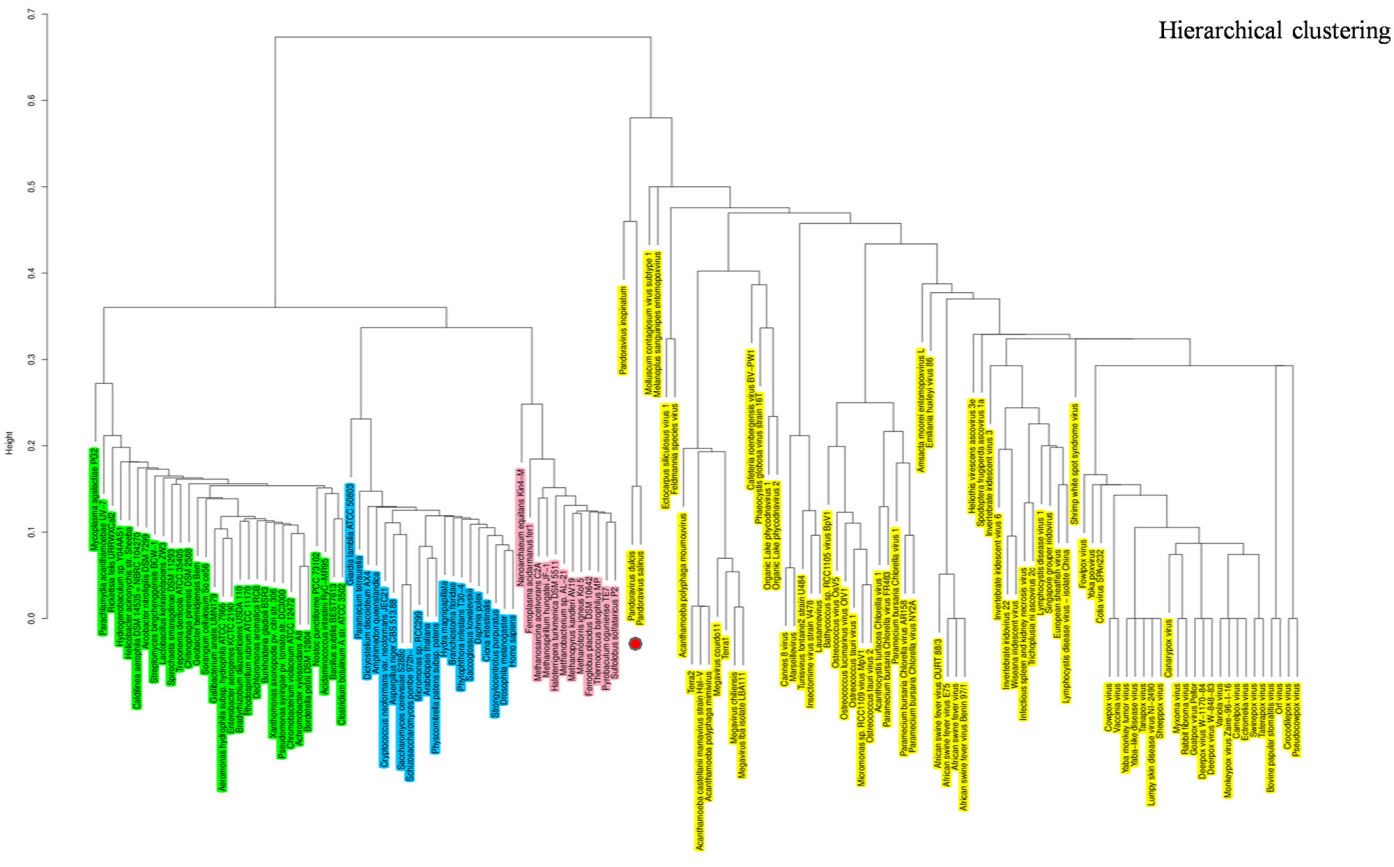
**Hierarchical clustering by phyletic pattern.** Dendrogram was constructed based on the presence/absence of informational clusters of orthologous groups of proteins (COGs) in *Megavirales* (yellow), *Bacteria* (green), *Archaea* (pink), *and Eukarya* (blue).

The hierarchical clustering dendrogram based on the presence/absence matrix using 737 COGs comprised of informational genes sharply delineated four clades. Pandoraviruses were embedded in the *Megavirales* branch. This finding is consistent with those from phylogenetic analyses, indicating that pandoraviruses are new genuine *Megavirales* members (**Figure 6**). Six COGs were present in *P. salinus* and *P. dulcis* and absent in other megaviruses. These COGs are widespread throughout the genomes of the members of the three cellular TRUCs, and only three of these genomes harbor less than two of these COGs and are considered to be short-sized genomes [491–1,485 kilobase pairs (kbp)]. Conversely, 70 COGs were absent from the *P. salinus* and *P. dulcis* genomes but present in at least one other *Megavirales* member, and these COGs were more abundant among mimiviruses, and secondarily, marseilleviruses, and phycodnaviruses. In addition, these COGs are widely represented in cellular organisms, the lowest number being 24. Moreover, the genome of *P. inopinatum* encodes 32 COGs among which 14 are shared with *P. dulcis* and 10 with *P. salinus*. Consistently, phyletic analyses considering similar gene contents and sizes showed that pandoraviruses clustered with *Megavirales*.

### Comparative analysis of *Pandoravirus* genomes

Comparative analyses performed using OrthoMCL with gene repertoires from *P. salinus* (2,556 predicted genes), *P. dulcis* (1,502 predicted genes; Philippe et al., 2013) and *P. inopinatum* (1,902 predicted genes; Antwerpen et al., 2015) identified 1,103 COGs, out of which 681 were core genes, being shared by all three pandoraviruses. In addition, *P. inopinatum* was found to share 179 genes with *P. salinus* and 73 with *P. dulcis*. Besides, the number of pairs of genes involved in reciprocal best BLASTp hits was 562 for *P. salinus* and *P. inopinatum*, 510 for *P. dulcis* and *P. inopinatum,* and 668 for *P. salinus* and *P. dulcis*; while 375 of these *bona fide* orthologs shared by *P. salinus* and *P. dulcis* were also shared by these two viruses and *P. inopinatum*.

## Discussion

Herein, we used the informational genes shared by *Megavirales* members and cellular organisms to demonstrate that *P. salinus, P. dulcis* and *P. inopinatum* are three new genuine members of the fourth TRUC. Thus, these giant viruses, the largest identified so far, were clustered with other megaviruses based on both phylogenetic and phyletic analyses conducted using a previously published strategy, which showed that *Megavirales* members comprise a monophyletic group apart from *Bacteria*, *Archaea,* and *Eukarya* (Boyer et al., 2010). In addition, in the present study, we used relevant criteria for the selection of cellular organisms, as recently reported (Sharma et al., 2014), to obtain a better understanding of the origin and evolution of *Megavirales* members.

RNA polymerase 1 and 2 subunits are universal genes, conserved in giant viruses and cellular organisms. Moreover, these genes are refractory to lateral gene transfer and, in viruses, these genes are resistant to recombination with the host genome (Case et al., 2007; Adékambi et al., 2009). The ubiquity of RNAP facilitated the construction of a phylogenetic tree comprising all studied organisms, confirming that pandoraviruses, and other giant viruses, comprised a fourth branch in the tree of life (Boyer et al., 2010; Sharma et al., 2014). Moreover, the phylogenies of other informational genes (including DNApol and TFIIB) with vertical evolution and poor evidence of recombination established a clear evolutionary picture, in which pandoraviruses, and other giant viruses, are new members of a ‘Fourth TRUC’ club. Phylogenetic analyses based on these genes and phyletic analysis using a set of informational COGs revealed that *Megavirales* members comprise a monophylogenetic super phylum, consistent with previous findings (Boyer et al., 2010; Colson et al., 2011). It has been suggested that *Megavirales* representatives evolved a majority of their genes via horizontal gene transfer and therefore do not represent a fourth TRUC (Williams et al., 2011). In contrast, phylogenomic analyses using conserved structural domains have also revealed large to medium DNA viruses as ancient biological entities distinct from *Bacteria*, *Archaea,* and *Eukarya* (Nasir et al., 2012). We also conducted a phylogenetic analysis based on RNR small subunit, which plays an important role in nucleotide metabolism, and an aminoacyl-tRNA synthetase, and the results suggested complex evolutionary histories with multiple horizontal gene transfers among life forms from different TRUC, as previously reported (Wolf et al., 1999; Moreira et al., 2005; Lundin et al., 2010).

Previous studies have suggested that *Megavirales* members originate from an ancestral virus, and phylogenetic analyses based on certain informational genes represent these organisms apart from *Bacteria*, *Archaea,* and *Eukarya* (Yutin et al., 2009; Boyer et al., 2010; Koonin and Yutin, 2010; Colson et al., 2012; Yutin and Koonin, 2013; Sharma et al., 2014). The finding that phylogenetic reconstructions, based on RNAP1 and RNAP2 genes, display the same topologies, and are consistent with the ribosomal tree of life, suggests that both genes followed the same monophylogenetic evolutionary history. The phylogenetic and phyletic analyses performed herein obtained the same results in favor of *Megavirales* as a fourth super-group, supporting the results of previous studies, and provide insight into the vertical and horizontal evolution of pandoraviruses.

Pandoraviruses are mosaic organisms, similar to other microbes, and they are highly divergent from other *Megavirales* members. A small proportion of the pandoravirus gene complement is indeed shared with other known giant viruses, and these organisms even lack a majority of the *Megavirales* core genes (Philippe et al., 2013), while only five of the 12 informational genes previously identified by Boyer et al. (2010) were detected in the *Pandoravirus* genome. Notwithstanding, some genes allow the construction of phylogenies showing *Megavirales* as a monophyletic group and suggest that early representatives may have harbored these *bona fide* ancient genes, then lost genes and acquired new ones from their hosts and other sources. Moreover, phylogenetic analyses based on genes with vertical evolution and phyletic analysis of the presence/absence of conserved informational COGs strongly suggest that pandoraviruses are new genuine members of the ‘Fourth TRUC’ club. Interestingly, pandoraviruses were found to be taxonomically the closest to coccolithoviruses, which belong to the family *Phycodnaviridae*. This is congruent with results from previous studies obtained using genes from *P. salinus* and *P. dulcis* (Philippe et al., 2013; Yutin and Koonin, 2013). It has been further hypothesized that pandoraviruses were highly derived phycodnaviruses that might have experienced considerable genome expansion from smaller *Megavirales* representatives (Yutin and Koonin, 2013).

## Conclusion

Phylogenetic and phyletic analyses using informational genes suggest that pandoraviruses are new *bona fide* members of the ‘Fourth TRUC’ club, in addition to newly sequenced *Megavirales* representatives. The monophylogeny of *Megavirales* suggests a common origin and evolution from ancestral DNA viruses.

## Conflict of Interest Statement

The authors declare that the research was conducted in the absence of any commercial or financial relationships that could be construed as a potential conflict of interest.

## Footnotes

1 ftp://ftp.ncbi.nih.gov/genomes/Viruses/

2 http://www.ncbi.nlm.nih.gov/genbank/

3 http://tree.bio.ed.ac.uk/software/figtree/

4 http://www.r-project.org/

## References

[B1] Adékambi T. Drancourt M. Raoult D. (2009). The rpoB gene as a tool for clinical microbiologists. *Trends Microbiol.* 17 37–45 10.1016/j.tim.2008.09.008 19081723

[B2] Aherfi S. Boughalmi M. Pagnier I. Fournous G. La Scola B. Raoult D. (2014). Complete genome sequence of Tunisvirus, a new member of the proposed family Marseilleviridae. *Arch. Virol.* 159 2349–2358 10.1007/s00705-014-2023-5 24770845

[B3] Altschul S. F. Gish W. Miller W. Myers E. W. Lipman D. J. (1990). Basic local alignment search tool. *J. Mol. Biol.* 215 403–410 10.1016/S0022-2836(05)80360-2 2231712

[B4] Antwerpen M. H. Georgi E. Zoeller L. Woelfel R. Stoecker K. Scheid P. (2015). Whole-genome sequencing of a *Pandoravirus* isolated from keratitis-inducing *Acanthamoeba*. *Genome Announc.* 3:e00136-15. 10.1128/genomeA.00136-15 PMC4384135 25814595

[B5] Arslan D. Legendre M. Seltzer V. Abergel C. Claverie J.-M. (2011). Distant Mimivirus relative with a larger genome highlights the fundamental features of Megaviridae. *Proc. Natl. Acad. Sci. U.S.A.* 108 17486–17491 10.1073/pnas.1110889108 21987820 PMC3198346

[B6] Boughalmi M. Pagnier I. Aherfi S. Colson P. Raoult D. La Scola B. (2013). First isolation of a marseillevirus in the diptera syrphidae eristalis tenax. *Intervirology* 56 386–394 10.1159/000354560 24157885

[B7] Boyer M. Madoui M.-A. A. Gimenez G. La Scola B. Raoult D. (2010). Phylogenetic and phyletic studies of informational genes in genomes highlight existence of a 4th domain of life including giant viruses. *PLoS ONE* 5:e15530 10.1371/journal.pone.0015530 PMC2996410 21151962

[B8] Boyer M. Yutin N. Pagnier I. Barrassi L. Fournous G. Espinosa L. (2009). Giant Marseillevirus highlights the role of amoebae as a melting pot in emergence of chimeric microorganisms. *Proc. Natl. Acad. Sci. U.S.A.* 106 21848–21853 10.1073/pnas.0911354106 20007369 PMC2799887

[B9] Case R. J. Boucher Y. Dahllöf I. Holmström C. Doolittle W. F. Kjelleberg S. (2007). Use of 16S rRNA and rpoB genes as molecular markers for microbial ecology studies. *Appl. Environ. Microbiol.* 73 278–288 10.1128/AEM.01177-06 17071787 PMC1797146

[B10] Colson P. De Lamballerie X. Fournous G. Raoult D. (2012). Reclassification of giant viruses composing a fourth domain of life in the new order *Megavirales*. *Intervirology* 55 321–332 10.1159/000336562 22508375

[B11] Colson P. De Lamballerie X. Yutin N. Asgari S. Bigot Y. Bideshi D. K. (2013). “*Megavirales*,” a proposed new order for eukaryotic nucleocytoplasmic large DNA viruses. *Arch. Virol.* 158 2517–2521 10.1007/s00705-013-1768-6 23812617 PMC4066373

[B12] Colson P. Gimenez G. Boyer M. Fournous G. Raoult D. (2011). The giant cafeteria roenbergensis virus that infects a widespread marine phagocytic protist is a new member of the fourth domain of life. *PLoS ONE* 6:e18935 10.1371/journal.pone.0018935 PMC3084725 21559486

[B13] Crona M. Avesson L. Sahlin M. Lundin D. Hinas A. Klose R. (2013). A rare combination of ribonucleotide reductases in the social amoeba *Dictyostelium discoideum*. *J. Biol. Chem.* 288 8198–8208 10.1074/jbc.M112.442434 23372162 PMC3605638

[B14] Dixon L. K. Costa J. V. Escribano J. M. Rock D. L. Vinuela E. Wilkinson P. J. (2000). “Family asfarviridae,” in *Virus taxonomy. Seventh report of the International Committee on Taxonomy of Viruses* eds van Regenmortel M. H. V. Fauquet C. M. Bishop D. H. L. Carstens E. B. Estes M. K. Lemon S. M. (San Diego, CA: Academic Press) 159–165.

[B15] Edgar R. C. (2004). MUSCLE: a multiple sequence alignment method with reduced time and space complexity. *BMC Bioinform.* 5:113 10.1186/1471-2105-5-113 PMC517706 15318951

[B16] Fischer M. G. Allen M. J. Wilson W. H. Suttle C. A. (2010). Giant virus with a remarkable complement of genes infects marine zooplankton. *Proc. Natl. Acad. Sci. U.S.A.* 107 19508–19513 10.1073/pnas.1007615107 20974979 PMC2984142

[B17] Ghedin E. Claverie J.-M. (2005). Mimivirus relatives in the Sargasso sea. *Virol. J.* 2 62 10.1186/1743-422X-2-62 PMC1215527 16105173

[B18] Hedges S. B. Dudley J. Kumar S. (2006). TimeTree: a public knowledgebase of divergence times among organisms. *Bioinformatics* 22 2971–2972 10.1093/bioinformatics/btl505 17021158

[B19] Huang Y. Niu B. Gao Y. Fu L. Li W. (2010). CD-HIT Suite: a web server for clustering and comparing biological sequences. *Bioinformatics* 26 680–682 10.1093/bioinformatics/btq003 20053844 PMC2828112

[B20] Iyer L. M. Aravind L. Koonin E. V. (2001). Common origin of four diverse families of large eukaryotic DNA viruses. *J. Virol.* 75 11720–11734 10.1128/JVI.75.23.11720-11734.2001 11689653 PMC114758

[B21] Jordan I. K. Rogozin I. B. Wolf Y. I. Koonin E. V. (2002). Essential genes are more evolutionarily conserved than are non-essential genes in bacteria. *Genome Res.* 12 962–968 10.1101/gr.87702 12045149 PMC1383730

[B22] Kerepesi C. Grolmusz V. (2015). Nucleotide sequences of giant viruses found in soil samples of the Mojave desert, the prairie, the tundra and the Antarctic dry valleys. *arXiv:*1503.05575v1. 10.1126/science.1101485.31.48

[B23] Koonin E. V. Yutin N. (2010). Origin and evolution of eukaryotic large nucleo-cytoplasmic DNA viruses. *Intervirology* 53 284–292 10.1159/000312913 20551680 PMC2895762

[B24] Kristensen D. M. Mushegian A. R. Dolja V. V. Koonin E. V. (2010). New dimensions of the virus world discovered through metagenomics. *Trends Microbiol*. 18 11–19 10.1016/j.tim.2009.11.003 19942437 PMC3293453

[B25] La Scola B. Desnues C. Pagnier I. Robert C. Barrassi L. Fournous G. (2008). The virophage as a unique parasite of giant Mimivirus. *Nature* 455 100–104 10.1038/nature07218 18690211

[B26] Li L. L. Stoeckert C. J. Roos D. S. (2003). OrthoMCL: identification of ortholog groups for eukaryotic genomes. *Genome Res.* 13 2178–2189 10.1101/gr.1224503 12952885 PMC403725

[B27] Lundin D. Gribaldo S. Torrents E. Sjöberg B.-M. Poole A. M. (2010). Ribonucleotide reduction - horizontal transfer of a required function spans all three domains. *BMC Evol. Biol.* 10:383 10.1186/1471-2148-10-383 PMC3019208 21143941

[B28] Monier A. Claverie J.-M. Ogata H. (2008). Taxonomic distribution of large DNA viruses in the sea. *Genome Biol.* 9 R106. 10.1186/gb-2008-9-7-r106 PMC2530865 18598358

[B29] Moreira D. Brochier-Armanet C. (2008). Giant viruses, giant chimeras: the multiple evolutionary histories of Mimivirus genes. *BMC Evol. Biol.* 8:12 10.1186/1471-2148-8-12 PMC2263039 18205905

[B30] Moreira D. López-García P. Ogata H. Abergel C. Raoult D. Claverie J. M. (2005). Comment on the 1.2-megabase genome sequence of Mimivirus. *Science* 308 1114 10.1126/science.1110820 15905382

[B31] Nasir A. Kim K. M. Caetano-Anolles G. (2012). Giant viruses coexisted with the cellular ancestors and represent a distinct supergroup along with superkingdoms Archaea, Bacteria and Eukarya. *BMC Evol. Biol.* 12:156 10.1186/1471-2148-12-156 PMC3570343 22920653

[B32] Pagnier I. Reteno D. G. I. Saadi H. Boughalmi M. Gaia M. Slimani M. (2013). A decade of improvements in mimiviridae and marseilleviridae isolation from amoeba. *Intervirology* 56 354–363 10.1159/000354556 24157882

[B33] Philippe N. Legendre M. Doutre G. Couté Y. Poirot O. Lescot M. (2013). Pandoraviruses: amoeba viruses with genomes up to 2.5 Mb reaching that of parasitic eukaryotes. *Science* 341 281–286 10.1126/science.1239181 23869018

[B34] Popgeorgiev N. Boyer M. Fancello L. Monteil S. Robert C. Rivet R. (2013). Marseillevirus-like virus recovered from blood donated by asymptomatic humans. *J. Infect. Dis.* 208 1042–1050 10.1093/infdis/jit292 23821720

[B35] Price M. N. Dehal P. S. Arkin A. P. (2010). FastTree 2–approximately maximum-likelihood trees for large alignments. *PLoS ONE* 5:e9490 10.1371/journal.pone.0009490 PMC2835736 20224823

[B36] Raoult D. (2013). TRUC or the need for a new microbial classification. *Intervirology* 56 349–353 10.1159/000354269 23867259

[B37] Raoult D. (2015). How the virophage compels the need to readdress the classification of microbes. *Virology* 477 119–124 10.1016/j.virol.2014.11.014 25497204

[B38] Raoult D. Audic S. Robert C. Abergel C. Renesto P. Ogata H. (2004). The 1.2-megabase genome sequence of Mimivirus. *Science* 306 1344–1350 10.1126/science.1101485 15486256

[B39] Raoult D. Forterre P. (2008). Redefining viruses: lessons from Mimivirus. *Nat. Rev. Microbiol.* 6 315–319 10.1038/nrmicro1858 18311164

[B40] Raoult D. La Scola B. Birtles R. (2007). The discovery and characterization of Mimivirus, the largest known virus and putative pneumonia agent. *Clin. Infect. Dis.* 45 95–102 10.1086/518608 17554709

[B41] Saadi H. Pagnier I. Colson P. Cherif J. K. Beji M. Boughalmi M. (2013). First isolation of Mimivirus in a patient with pneumonia. *Clin. Infect. Dis.* 57 e127–e134 10.1093/cid/cit354 23709652

[B42] Santini S. Jeudy S. Bartoli J. Poirot O. Lescot M. Abergel C. (2013). Genome of Phaeocystis globosa virus PgV-16T highlights the common ancestry of the largest known DNA viruses infecting eukaryotes. *Proc. Natl. Acad. Sci. U.S.A.* 110 10800–10805 10.1073/pnas.1303251110 23754393 PMC3696832

[B43] Scheid P. (2014). Relevance of free-living amoebae as hosts for phylogenetically diverse microorganisms. *Parasitol. Res.* 113 2407–2414 10.1007/s00436-014-3932-7 24828345

[B44] Scheid P. Balczun C. Schaub G. A. (2014). Some secrets are revealed: parasitic keratitis amoebae as vectors of the scarcely described pandoraviruses to humans. *Parasitol. Res.* 113 3759–3764 10.1007/s00436-014-4041-3 25033816

[B45] Scheid P. Hauröder B. Michel R. (2010). Investigations of an extraordinary endocytobiont in *Acanthamoeba* sp.: development and replication. *Parasitol. Res.* 106 1371–1377 10.1007/s00436-010-1811-4 20393749

[B46] Scheid P. Zöller L. Pressmar S. Richard G. Michel R. (2008). An extraordinary endocytobiont in *Acanthamoeba* sp. isolated from a patient with keratitis. *Parasitol. Res.* 102 945–950 10.1007/s00436-007-0858-3 18210154

[B47] Sharma V. Colson P. Giorgi R. Pontarotti P. Raoult D. (2014). DNA-dependent RNA polymerase detects hidden giant viruses in published databanks. *Genome Biol. Evol*. 6 1–22 10.1093/gbe/evu128 24929085 PMC4122926

[B48] Suttle C. A. (2005). Viruses in the sea. *Nature* 437 356–361 10.1038/nature04160 16163346

[B49] Thomas V. Bertelli C. Collyn F. Casson N. Telenti A. Goesmann A. (2011). Lausannevirus, a giant amoebal virus encoding histone doublets. *Environ. Microbiol.* 13 1454–1466 10.1111/j.1462-2920.2011.02446.x 21392201

[B50] Werner F. Grohmann D. (2011). Evolution of multisubunit RNA polymerases in the three domains of life. *Nat. Rev. Microbiol.* 9 85–98 10.1038/nrmicro2507 21233849

[B51] Williams T. A. Embley T. M. Heinz E. (2011). Informational gene phylogenies do not support a fourth domain of life for nucleocytoplasmic large DNA viruses. *PLoS ONE* 6:e21080 10.1371/journal.pone.0021080 PMC3116878 21698163

[B52] Williamson S. J. Allen L. Z. Lorenzi H. A. Fadrosh D. W. Brami D. Thiagarajan M. (2012). Metagenomic exploration of viruses throughout the indian ocean. *PLoS ONE* 7:e42047 10.1371/journal.pone.0042047 PMC3474794 23082107

[B53] Woese C. R. Kandler O. Wheelis M. L. (1990). Towards a natural system of organisms: proposal for the domains *Archaea, Bacteria, and Eucarya*. *Proc. Natl. Acad. Sci. U.S.A.* 87 4576–4579 10.1073/pnas.87.12.4576 2112744 PMC54159

[B54] Wolf Y. I. Aravind L. Grishin N. V. Koonin E. V. (1999). Evolution of aminoacyl-tRNA synthetases–analysis of unique domain architectures and phylogenetic trees reveals a complex history of horizontal gene transfer events. *Genome Res.* 9 689–710. 10447505

[B55] Wu D. Wu M. Halpern A. Rusch D. B. Yooseph S. Frazier M. (2011). Stalking the fourth domain in metagenomic data: searching for, discovering, and interpreting novel, deep branches in marker gene phylogenetic trees. *PLoS ONE* 6:e18011 10.1371/journal.pone.0018011 PMC3060911 21437252

[B56] Yoosuf N. Yutin N. Colson P. Shabalina S. A. Pagnier I. Robert C. (2012). Related giant viruses in distant locations and different habitats: *Acanthamoeba polyphaga* moumouvirus represents a third lineage of the Mimiviridae that is close to the *Megavirus lineage*. *Genome Biol. Evol.* 4 1324–1330 10.1093/gbe/evs109 23221609 PMC3542560

[B57] Yutin N. Colson P. Raoult D. Koonin E. V. (2013). Mimiviridae: clusters of orthologous genes, reconstruction of gene repertoire evolution and proposed expansion of the giant virus family. *Virol. J.* 10 106 10.1186/1743-422X-10-106 PMC3620924 23557328

[B58] Yutin N. Koonin E. V. (2012). Hidden evolutionary complexity of Nucleo-Cytoplasmic Large DNA viruses of eukaryotes. *Virol. J.* 9 161 10.1186/1743-422X-9-161 PMC3493329 22891861

[B59] Yutin N. Koonin E. V. (2013). Pandoraviruses are highly derived phycodnaviruses. *Biol. Dir.* 8 25 10.1186/1745-6150-8-25 PMC3924356 24148757

[B60] Yutin N. Wolf Y. I. Koonin E. V. (2014). Origin of giant viruses from smaller DNA viruses not from a fourth domain of cellular life. *Virology,* 466–467, 38–52 10.1016/j.virol.2014.06.032 PMC4325995 25042053

[B61] Yutin N. Wolf Y. I. Raoult D. Koonin E. V. (2009). Eukaryotic large nucleo-cytoplasmic DNA viruses: clusters of orthologous genes and reconstruction of viral genome evolution. *Virol. J.* 6 223 10.1186/1743-422X-6-223 PMC2806869 20017929

