# Supplementary figures and images for: Welcome to pandoraviruses at the ‘Fourth TRUC’ club
Source: Front Microbiol. 2015 May 18;6:423. doi: 10.3389/fmicb.2015.00423 (PMC4435241; doi:10.3389/fmicb.2015.00423)

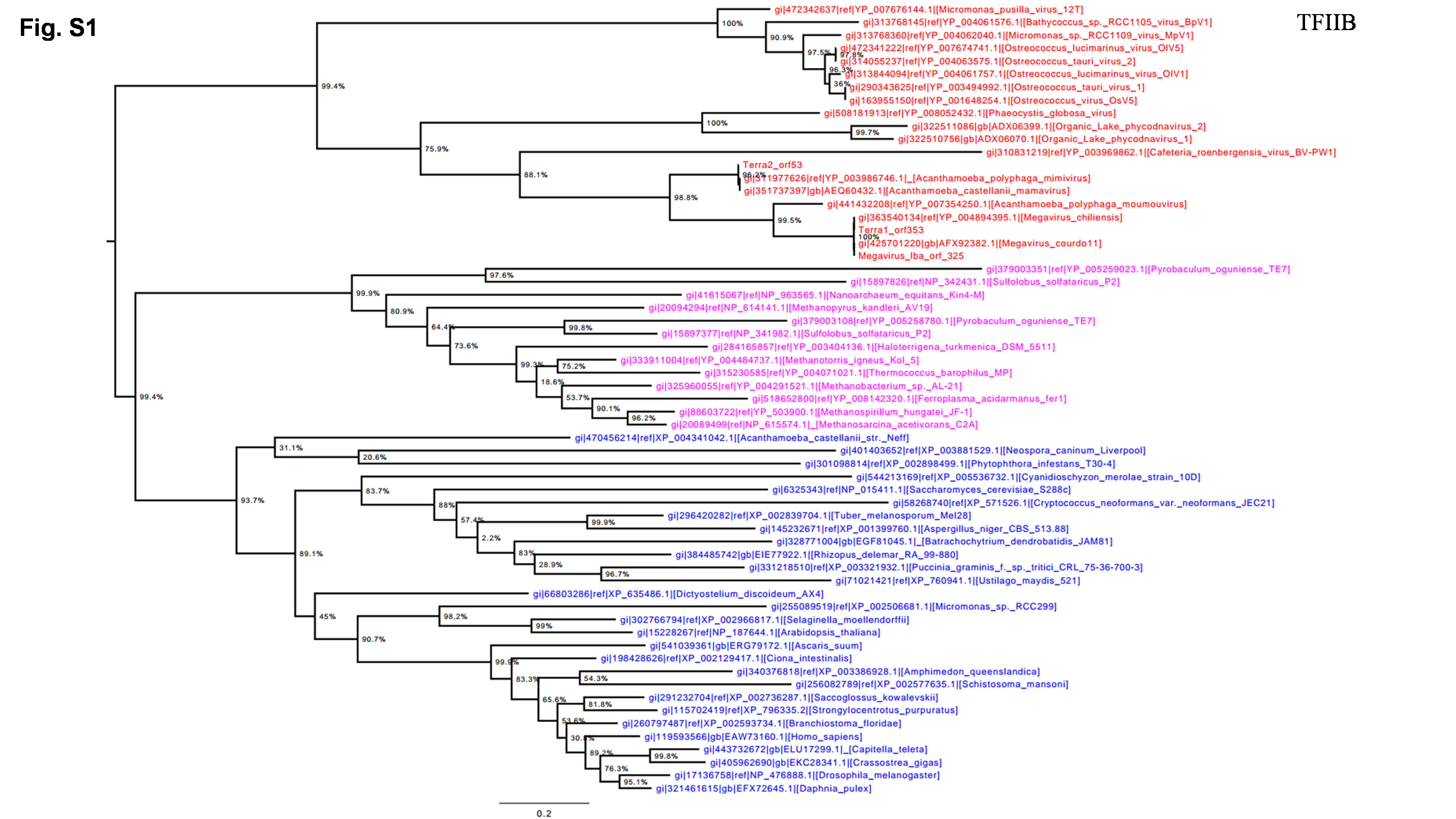

Supplement: Supplementary file 1 [file Image_1.TIF]
